# Inflammatory Myofibroblastic Tumor of the Lung: A Case Report

**DOI:** 10.7759/cureus.70207

**Published:** 2024-09-25

**Authors:** Marianthi Baltagianni, Vasileios Leivaditis, Nikolaos Baltayiannis, Gabriela Stanc, Efstathia Souka, Pella Batika, Eleftherios Beltsios, Francesk Mulita, Athanasios Papatriantafyllou, Efstratios N Koletsis

**Affiliations:** 1 Department of Thoracic Surgery, Metaxa Cancer Hospital, Piraeus, GRC; 2 Department of Cardiothoracic and Vascular Surgery, Westpfalz-Klinikum, Kaiserslautern, DEU; 3 Department of Pathology, Metaxa Cancer Hospital, Piraeus, GRC; 4 Department of Anesthesiology and Critical Care, Hannover Medical School, Hannover, DEU; 5 Department of Cardiothoracic Surgery, General University Hospital of Patras, Patras, GRC; 6 Department of Surgery, General University Hospital of Patras, Patras, GRC

**Keywords:** inflammation, inflammatory myofibroblastic tumor, lung tumor, mesenchymal tumor, thoracic

## Abstract

Inflammatory myofibroblastic tumor (IMT) is a rare mesenchymal tumor classified as an intermediate malignancy that rarely metastasizes. The most common site for IMTs is the lung, though they can develop in various other anatomical locations. Pulmonary IMTs are more common in children and adolescents and are infrequently diagnosed in adults. This case report describes a 49-year-old woman with a history of breast cancer previously treated with subtotal mastectomy and chemotherapy who developed an IMT in the lung. This case emphasizes the rarity of the disease and the associated clinical challenges when managing such cases.

## Introduction

Inflammatory myofibroblastic tumor (IMT), first described as an inflammatory pseudotumor in 1903, was initially noted as a benign, non-infectious lesion primarily affecting the periocular region [1]. Characterized by a proliferation of fibroblasts and myofibroblasts with accompanying chronic inflammatory infiltration by lymphocytes, plasma cells, eosinophils, and mast cells, it was redefined in 1990 as a distinct pathological entity from the broader category of inflammatory pseudotumors [2]. IMTs are now recognized as rare neoplasms, having a prevalence ranging from 0.04% to 0.7%, with an intermediate degree of malignancy, and are known for their tendency to recur, with recurrence rates of up to 25%, but rarely metastasizing, with a less than 5% chance [2,3].

These tumors can occur in virtually any anatomical location, including the mesentery, gastrointestinal tract, retroperitoneum, mediastinum, heart, soft tissues, larynx, uterus, bones, and central nervous system [4,5]. However, the lung is the most common site for IMTs, where they are typically located peripherally, mainly in the lower lobes [6]. The intrabronchial localization of these tumors is exceedingly rare, comprising less than 1% of cases. The etiology and pathogenesis of IMTs remain unclear, with potential contributing factors including inflammation, trauma, autoimmune diseases, previous surgery, viral infections, and abnormal healing with uncontrolled myofibroblast proliferation [7].

IMTs must be distinguished from other entities, such as epithelial inflammatory myofibroblast sarcoma (EIMS), which follows a more aggressive clinical course with a high mortality rate [8]. The primary treatment for IMTs is complete surgical resection, which is associated with long-term survival and a significantly reduced recurrence rate [9]. Chemotherapy is generally not recommended as an adjuvant treatment but may be necessary in cases where complete resection is unachievable [10].

In this report, we present the case of a 49-year-old woman with a history of breast cancer who developed an IMT in the lung; we also review the relevant literature to provide a comprehensive overview of this rare clinical entity.

## Case presentation

**Figure 1:**
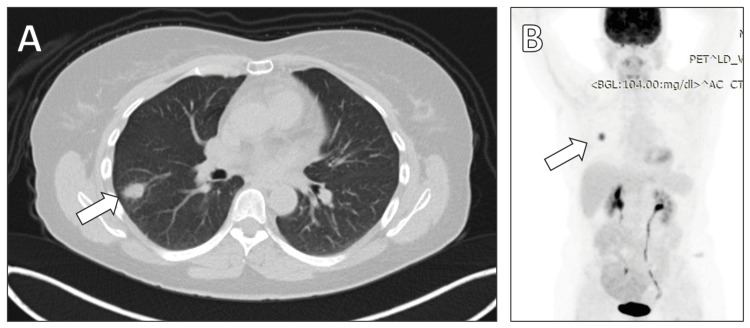
Preoperative diagnostic imaging (A) A computed tomography (CT) scan of the chest showing a neoplastic mass in the right lower lobe of the lung. (B) Positron emission tomography/CT (PET/CT) imaging of the lung neoplasm with a maximum standardized uptake value (SUVmax) of 8.2, indicating high metabolic activity.

A 49-year-old woman with a known history of breast cancer, previously treated with subtotal mastectomy and chemotherapy, presented for routine follow-up. A recent computed tomography (CT) scan revealed a mass in the right lower lobe of the lung (Figure 1A). Further evaluation with positron emission tomography/CT (PET/CT) imaging showed a positive maximum standardized uptake value (SUVmax) of 8.2 (Figure 1B).

The patient underwent a complete clinical and laboratory workup, including blood tests, cardiopulmonary assessment, and spirometry. The measured laboratory parameters were in the normal range and are presented in Table 1.

**Table 1:** Laboratory blood test values of the patient GOT = glutamic-oxaloacetic transaminase, AST = aspartate aminotransferase, GPT = glutamic-pyruvic transaminase, ALT = alanine aminotransferase, gamma-GT = gamma-glutamyl transferase, LDH = lactate dehydrogenase, CK = creatine kinase, ALP = alkaline phosphatase, BUN = blood urea nitrogen, GFR = glomerular filtration rate, CRP = C-reactive protein, WBC = white blood cell, RBC = red blood cell, Hb = hemoglobin, Ht = hematocrit, PLT = platelet

Parameter	Value	Normal Range
Natrium	142	136-145 mmol/l
Potassium	3.8	3.4-4.5 mmol/l
GOT/AST	28	10-35 U/l
GPT/ALT	23	10-35 U/l
Gamma-GT	16	<40 U/l
LDH	134	<250 U/l
CK	62	20-180 U/l
ALP	73	35-104 U/l
BUN	39	16.6-48.5 mg/dl
Creatinine	1.02	0.50-0.90 mg/dl
GFR	145	>90 ml/min/1.73 m^2^
Bilirubin	1.13	0-1.20 mg/dl
Glucose	87	60-99 mg/dl
CRP	12.3	<5 mg/dl
WBC	11.34	4.0-11.2 × 10^3^/µl
RBC	4.7	4.1-5.4 × 10^6^/µl
Hb	12.6	11.5-16.0 g/dl
Ht	38.2	36.0%-48.0%
PLT	236	176-391 × 10^3^/µl

The measured spirometry values are demonstrated in Table 2.

**Table 2:** Spirometry values of the patient The parameters include measured values, expected values based on population norms, the percentage of the measured value relative to the expected value, and the normal range for each parameter. FVC = forced vital capacity, FEV_1_ = forced expiratory volume in one second, PEF = peak expiratory flow, TLC = total lung capacity

Parameter	Measured Value	Expected Value	Percentage	Normal Range
FVC (l)	3.1	3.5	88	3.0-4.0
FEV_1_ (l)	2.9	3.0	97	2.5-3.2
FEV_1_/FVC (%)	78	80	97.5	75%-80%
PEF (l/min)	450	480	93.75	300-500
TLC (l)	5.5	5.8	94.83	4.7-6.0

A histological diagnosis was pursued, and the patient underwent a CT-guided fine needle biopsy (FNB) of the lung mass. The pathological report confirmed the diagnosis of an inflammatory myofibroblastic tumor. Following multidisciplinary team discussions, it was decided to proceed with surgical resection of the neoplasm. The patient underwent a limited right thoracotomy and standard right lower lobectomy with lymph node dissection. An R0 resection with free surgical margins was achieved. The size of the tumor was measured at 2.3 x 1.6 x 1.9 cm.

**Figure 2:**
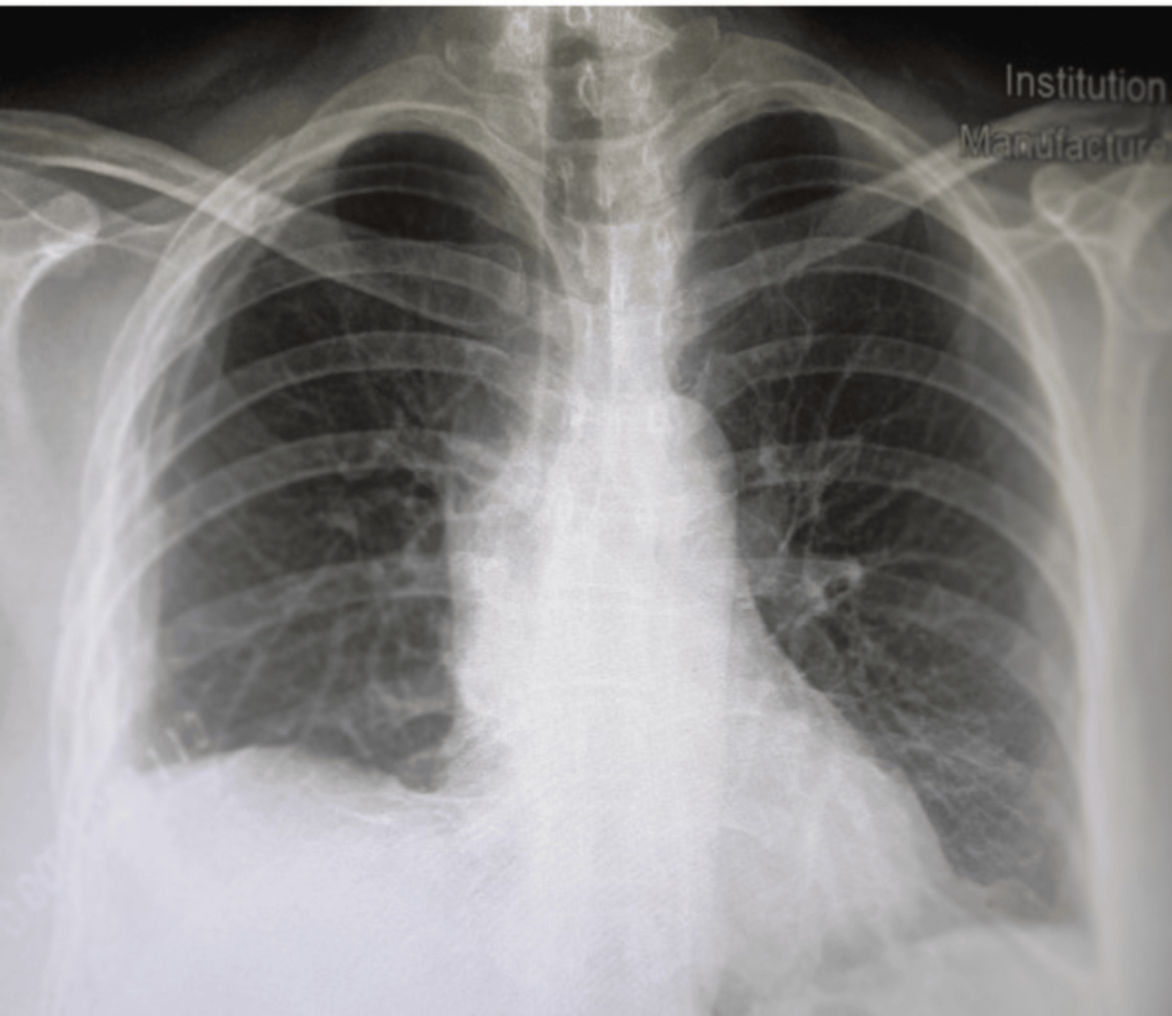
Postoperative imaging A chest radiograph from postoperative day 2 demonstrating the satisfactory position of the Bülau drainage tube in the right hemithorax.

Postoperatively, the patient had an uneventful recovery and was discharged on the fifth postoperative day in an excellent condition (Figure 2).

**Figure 3:**
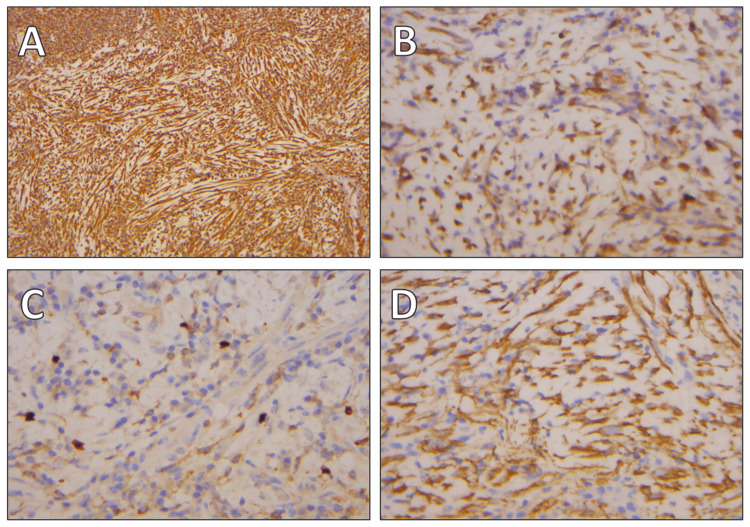
Immunohistochemical studies (A) Vimentin positive staining at 100x magnification. (B) Calponin positive staining at 400x magnification. (C) Smooth muscle actin (SMA) positive staining at 400x magnification. (D) Ki-67 staining at 400x magnification, indicating a proliferation index of 1%-5% in neoplastic cells.

The final histopathological examination confirmed the diagnosis of the IMT, showing tractiform cells growing in a myofibromatous layer consisting of interlaced bundles. Immunohistochemically, the tumor cells were positive for vimentin, calponin, desmin, and ALK, with a proliferation index (Ki-67) of 1%-5% (Figure 3).

The neoplastic cells did not exhibit significant atypia or mitotic activity. Regular follow-ups were recommended for monitoring both the IMT and the previous breast cancer.

## Discussion

The occurrence of multiple primary cancers, as first defined by Warren and Gates in 1932, requires the identification of at least two malignant tumors in a single patient, histological confirmation of malignancy, and the exclusion of metastatic spread [11]. According to Moertel's criteria, if multiple primary cancers are diagnosed within six months, they are considered concurrent, whereas diagnoses made more than six months apart are considered late [12]. In this case, the IMT was classified as a late neoplasm.

WHO officially recognized IMTs as a distinct pathological entity in 1994. The simultaneous occurrence of multiple primary cancers and IMTs is exceedingly rare [13]. While IMTs can develop in almost any anatomical region, their presentation in the lung is particularly notable. Pulmonary IMTs are more common in children and adolescents and are typically located in the parenchyma rather than appearing endobronchially [14]. This case underlines the importance of considering IMTs in the differential diagnosis of lung masses, especially in patients with a history of malignancy.

The clinical presentation of IMTs varies depending on the tumor's location. Common symptoms include fever, weight loss, and malaise, alongside local signs and symptoms related to the tumor's mass effect and reactive inflammation [5]. In the lung, IMTs can cause respiratory symptoms such as cough, dyspnea, and hemoptysis. Imaging studies, including CT and MRI, typically reveal a heterogeneous or homogeneous mass characterized by hyper- or hypovascularity, with or without calcifications [15]. In this case, the IMT was detected incidentally during a routine follow-up imaging for the patient's previous breast cancer.

The primary treatment for IMTs is complete surgical resection, which offers the best chance for long-term survival and reduces the recurrence rate to approximately 2% [9]. Achieving complete (R0) resection can be challenging, with reported success rates ranging from 72% to 89% [16]. In cases where complete resection is not feasible, adjuvant therapies, including chemotherapy and targeted treatments, may be employed [10]. Targeted therapies, such as tyrosine kinase inhibitors (TKIs) directed against ALK, ROS1, and NTRK1/3 fusion proteins, have shown promise in the management of unresectable or metastatic IMTs [17]. In this case, the patient's IMT was successfully resected, and no adjuvant therapy was deemed necessary.

The pathogenesis of IMTs remains poorly understood, with various factors implicated, including inflammation, trauma, autoimmune diseases, previous surgery, viral infections, and abnormal healing with uncontrolled myofibroblast proliferation [7]. Advances in next-generation sequencing (NGS) and computer-aided histopathological diagnosis have provided deeper insights into the molecular biology of IMTs, aiding in more precise diagnostic and therapeutic strategies [18].

Regular follow-up is crucial for patients with IMTs due to the potential for recurrence, even after complete surgical resection. Long-term monitoring with imaging studies and clinical evaluations is recommended to detect any signs of recurrence or progression [19]. In this case, the patient was advised to undergo regular follow-ups for both the IMT and the previous breast cancer to ensure early detection of any recurrence or new primary malignancies.

## Conclusions

This case report highlights the rare occurrence of an inflammatory myofibroblastic tumor in an adult with a history of breast cancer. The successful surgical resection of the tumor underlines the importance of a multidisciplinary approach in the management of such complex cases. Long-term follow-up remains crucial due to the potential for recurrence. Advances in diagnostic and therapeutic modalities, including NGS and targeted therapies, hold promise for improving the management of IMTs.
